# Addressing Childhood Obesity in Children in Need in Greece: Policy Implementers’ Knowledge, Perceptions and Lessons for Effective Implementation

**DOI:** 10.3390/nu17162629

**Published:** 2025-08-14

**Authors:** Theodora Balafouti, Dimitra E. Strongylou, Vaios Svolos, Matzourana Argyropoulou, Renos Roussos, Christina Mavrogianni, Alexios Manidis, Anela Halilagic, George Moschonis, Odysseas Androutsos, Yannis Manios, Theodora Mouratidou

**Affiliations:** 1 Department of Nutrition and Dietetic Sciences, School of Health Sciences, Hellenic Mediterranean University, 72300 Sitia, Greece; tbalafouti@hmu.gr (T.B.); royssos_r@outlook.com (R.R.); myx47@edu.hmu.gr (A.M.); 2 Lab of Clinical Nutrition and Dietetics, Department of Nutrition and Dietetics, School of Physical Education, Sports Science and Dietetics, University of Thessaly, 42100 Trikala, Greece; ntemi_st@hotmail.com (D.E.S.); vaiossvolos@gmail.com (V.S.); oandroutsos@uth.gr (O.A.); 3 School of Medicine, Dentistry and Nursing, College of Medical, Veterinary and Life Sciences, University of Glasgow, Glasgow G12 8QF, UK; 4 Department of Nutrition and Dietetics, School of Health Sciences and Education, Harokopio University of Athens, Kallithea, 17671 Athens, Greece; margyropoulou@hua.gr (M.A.); cmavrog@hua.gr (C.M.); manios@hua.gr (Y.M.); 5 Department of Food, Nutrition and Dietetics, School Allied Health, Human Services & Sport, La Trobe University, Melbourne, VIC 3086, Australia; a.halilagic@latrobe.edu.au (A.H.); g.moschonis@latrobe.edu.au (G.M.); 6 La Trobe Institute for Sustainable Agriculture & Food (LISAF), La Trobe University, Melbourne, VIC 3086, Australia; 7 Institute of Agri-Food and Life Sciences, University Research & Innovation Center, (H.M.U.R.I.C.), Hellenic Mediterranean University, 71003 Hellenic, Greece

**Keywords:** childhood obesity, regulatory framework, experiences and perceptions

## Abstract

**Background/Objectives:** Policy implementers play a crucial role in the effective delivery of policies aiming at promoting a healthy lifestyle in the most vulnerable populations. This study aimed to explore (a) policy implementers’ knowledge and perceptions of the policy framework promoting physical activity and healthy nutrition among children in need in Greece, and (b) self-perceived barriers and facilitators of the framework implementation. The term children in need refers to children who are at risk of poverty and/or social exclusion. **Methods:** A qualitative study design was employed consisting of semi-structured interviews with 25 policy implementers, who represented four delivery systems (health, social protection, food, and education sectors) from three geographical regions in Greece. Interviews were completed between November and December 2023. Thematic analysis was conducted using inductive and deductive approaches to identify key themes, following data management in the N-VIVO 14 software. **Results:** Commonly mentioned policies that study participants were involved in included school- and/or community-level-based behavioral interventions. Participants perceived policy implementation efforts that often relied on individual initiatives as inconsistent. Most participants argued that existing policies were not tailored to the needs of children in need. Major self-perceived barriers included limited personnel training, limited facilities and infrastructure, and lack of incentives or opportunities to encourage active participation. Major self-perceived facilitators included personnel motivation, integration of nutrition and physical education into school curricula, and provision of free school meals, which was associated with regular school attendance of children from the Roma communities. **Conclusions:** Individual, sociocultural, and structural issues are shown to persist across different delivery systems indicating the complexity of tackling obesogenic environments, especially among children in need. This is the first study in Greece to provide evidence on self-perceived barriers and facilitators and could inform ongoing national and European efforts to address obesogenic environments in children in need.

## 1. Introduction

Childhood obesity is a public health priority due to its high prevalence rate, in both developed and developing countries, and its association with short- and long-term negative health outcomes [1,2]. According to the World Health Organization (WHO), in 2022, around 37 million children < 5 years were living with overweight or obesity, while 390 million children aged 5–19 years were living with overweight, of which 160 million were living with obesity [3,4]. Greece has one of the highest prevalences of obesity in Europe (37.5% in children aged 2–14 years) [5]. According to recent UNICEF statistics, the rate of overweight and obesity in the country is double in children living in low-socioeconomic-status households [6]. Additionally, rates of overweight and obesity disproportionately affect culturally and linguistically diverse communities including vulnerable groups [7], accompanied by scarcity of evidence on lifestyle behaviors and their determinants and on best prevention practices for these groups [8,9,10].

In response to growing concerns, country- and/or region-specific prevention frameworks can serve as integral tools for tackling childhood obesity, especially in vulnerable groups. Such frameworks might include provision of healthy school meals, school canteen regulatory measures, and physical activity curricula [11]. A recent example of such efforts is the European Child Health Guarantee Strategy, which aims to address the social exclusion of children [12]. 

A recent scoping review mapping the regulatory and operational framework in Greece indicated limited information of the effectiveness, implementation, and impact of interventions tackling childhood obesity in children in need [11]. Notably, the review identified these children as the most understudied population group, with only 1 intervention, out of the 28, targeting them. However, it is worth noting that Greece has made substantial progress, implementing several regulatory frameworks to address childhood obesity, including the most recent National Action against Childhood Obesity (2023–2025) [6]. It is generally agreed that the problem of childhood obesity is highly complex, with recent evidence suggesting that multi-component, multi-disciplinary, and multi-setting interventions are needed to address the issue effectively, including associated implementation concerns [13].

Policy implementers play a vital role in the delivery of policies and are considered the “gatekeepers” of addressing implementation challenges. Understanding their experiences regarding barriers and facilitators involved in the implementation process is essential for effective prevention efforts. The results of a study on the implementation process of government-led food policies identified several facilitators, including leadership, effective resource utilization, supportive organizational structure, and presence of a monitoring and accountability system. Conversely, weak policy commitment and inadequate governance were cited as major barriers [14]. In terms of childhood obesity prevention policies, the results of a survey, with 187 policymakers and stakeholders across 12 EU Member States, identified several facilitators for successful program implementation including a supportive environment promoting physical activity and healthy nutrition, beneficiaries’ positive attitude towards the policy, school staff governance, and marketing restrictions in schools. Lack of motivation and support from school staff and a lack of supportive environment promoting physical activity were important barriers [15]. Similarly, results from an Australian study including 49 service health and wellbeing providers indicated that low participation of culturally and linguistically diverse migrant groups was due to community- and service-level barriers. The results highlighted the need of incorporating cultural competency training to service providers involved in the delivery of prevention strategies [7]. Elsewhere, it was noted that social vulnerabilities and inequalities could jeopardize the effectiveness of childhood obesity prevention strategies [16].

As such, this study was aimed at exploring (a) policy implementers’ knowledge and perceptions regarding the policy framework promoting physical activity and healthy nutrition in children in need in Greece, and (b) self-perceived barriers and facilitators of its implementation process.

## 2. Materials and Methods

### 2.1. Study Design

A cross-sectional multi-centered study design was employed. A qualitative study approach was used to explore and evaluate knowledge and perceptions of study participants. As described elsewhere [8], an authentic insight could only be achieved by endorsing an open qualitative approach given the novelty of the study and the scarcity of background data on the topic under investigation. The interviews were conducted between November and December 2023 in three diverse geographical regions of Greece, namely Attica (the region of the country’s capital city), Thessaly (a mid-size and centrally located region in Greece), and Crete (the biggest island in the Greece). Each selected region included rural and urban areas and parts with differential levels of deprivation.

### 2.2. Study Participants

Study participants were policy implementers, representing four key delivery systems (social protection, food, educational, and health systems), and were involved in the delivery of healthy eating and physical activity policies in children in need as part of their work. The identification term children in need refers to children who are at risk of poverty and/or social exclusion (i.e., homeless children or children experiencing severe housing deprivation; children with disabilities; children with mental health issues; children with a migrant background or minority ethnic origin, particularly Roma; children in alternative, especially institutional, care; and children in precarious family situations), and was used to recruit policy implementers involved in the implementation of related policy frameworks and delivery systems [12].

### 2.3. Procedure and Ethical Approval

The recruitment process involved email invitations to policy implementers. A convenient sampling approach was used to recruit participants, and all ethical procedures were followed, including the provision of an information sheet explaining the study aim, confidentiality issues, and participation details. Informed consent was obtained from all participants prior to the interviews, allowing adequate time for them to review the information sheet and address any queries regarding their participation. To maintain confidentiality, this study solely examined roles rather than the structures the participants represented. Individual face-to-face and online interviews (via video calls or teleconferences) were conducted by four experienced researchers (V.S., T.B., M.A., D.E.S.), who were trained prior to the data collection. Interviews, conducted in a private and quiet place, lasted between 60 and 75 min, and recordings were stored securely in an encrypted drive accessible only to the research team. The study received ethical approval from the Ethics Committee of Harokopio University (approval no: Γ-4081/06-10-2023), and the ethical guidelines of the Declaration of Helsinki were followed. 

### 2.4. Data Collection Approach and Materials

A semi-structured approach was employed, using open questions and allowing flexibility to adjust or skip questions based on the conversation flow. This approach allowed the exploration of key topics in depth, while enabling follow-up questions when participants gave important responses [17]. The topic guide was drafted by experts in childhood obesity based on existing scientific knowledge. A piloting process of the interview guide was carried out to assess clarity, relevance, and comprehensiveness by the research team and individuals without specialist knowledge. Questions focused on policy implementers’ knowledge and perceptions regarding the regulatory and operational framework addressing childhood obesity in Greece and its determinants, tailored to each of the four key delivery systems (Table S1a–d). A total number of 25 interviews were conducted to ensure a minimum representation from the three regions and key delivery systems. No new themes emerged in the final interviews, indicating that data saturation was achieved for the specific group of participants.

### 2.5. Data Analysis

Interview data were transcribed verbatim prior to analysis. The data collected was then anonymized and uploaded to N-VIVO 14 for analysis as described elsewhere [8]. Transcripts were coded using both inductive and deductive thematic analysis. Three researchers (T.B., R.R., D.E.M.) initially read and familiarized themselves with the transcripts and then generated preliminary codes using an inductive approach allowing themes to emerge from participants’ narratives. At the second stage, a deductive analysis was applied: codes were clustered into subthemes and broader themes, which were then mapped onto theoretical concepts drawn from the interview guide. This approach allowed for both data-driven coding and theory-based interpretation [18]. The researchers finalized the thematic categories for analysis once no new themes emerged. In the end, final themes, subthemes, and related quotes were transferred to an Excel file and translated into English. Discrepancies were discussed and resolved between researchers as part of data triangulation in order to enhance the credibility of the findings. To ensure the confidentiality of the interviewers, each participant was allocated with one unique code (identification number) that was used to reference their quotes.

## 3. Results

### 3.1. Participant Characteristics

Twenty-five policy implementers, from three distinct Greek geographical regions (Thessaly, Attica, and Crete), participated in the study, representing diverse national authorities and service provision structures (Table S2). Participants included special education teachers, physical educators, canteen managers, social workers, administrators in centers for refugee minors, cooks in child protection centers, and pediatricians. Table 1 describes the characteristics of the study participants. In summary, participants had a median age of 42 years and median job duration of 9 years. Most were females (72%) and had postgraduate degrees (68%). The distribution of implementers from the four delivery systems and the three geographical regions was equal in terms of their characteristics.

### 3.2. Themes

The themes derived from the analysis were classified into primary themes and subthemes and are presented below. The first theme refers to the policy implementers’ knowledge and experiences with existing policies addressing childhood obesity in Greece. The second theme refers to self-perceived determinants of policy implementation with subthemes including self-perceived barriers and facilitators. 

### 3.3. Theme 1: Policy Implementers’ Knowledge and Experiences with Existing Policies Promoting Physical Activity and Healthy Nutrition in Children in Need in Greece

All participants, across the four key delivery systems, reported being aware of, or not, of existing policies in their sector, with the majority reporting to be aware of their content. However, most implementers from the health sector stated that no such policies were in place in their sector.

*“The physical education textbook covers a wide range of activities, emphasizing that physical education extends beyond outdoor exercises.”*

(101)

*“When the state issues the legislation, it dιd not specify topics like vaccination, nutrition, exercise, or others. Instead, it leaves its implementation flexible, giving us the authority to decide what to prioritize and how to address these areas.”*

(221)

Nevertheless, some health sector implementers referred to broader service provision aiming at ensuring healthcare access for children in need including those from uninsured families.

*“We provide medical care to all children equally, whether they are disabled, Roma, refugees, even those who are uninsured or don’t have official documents.”*

(302)

Almost all participants reported knowledge or participation in policies or interventions promoting healthy nutrition and physical activity to children. The majority of the initiatives were community-driven and/or school-based, characterized by limited government involvement. Frequently mentioned school policies, among most participants, included “skill workshops”, physical education curricula in typical and special education schools, school canteen regulations, and food provision programs. Less mentioned school policies included nutrition modules in primary and secondary school curricula and vocational schools for children with disabilities. Most of the participants indicated breastfeeding promotion; nutrition policies in child protection and care units; food and material assistance for families; the National Dietary Guidelines for Infants, Children and Adolescents; and initiatives led by private organizations as common out-of-school policies. On the other hand, child protection measures like free access to sport centers, free meals, and financial support to vulnerable families were only reported by a small number of participants.

Additionally, some participants mentioned that there was no specific policy for children living with overweight or obesity, and that only few health professionals took the initiative to inform the parents and refer them for guidance to specialists.

*“A child living with obesity is weighed and measured, and if they fall outside the appropriate percentiles for their age, we refer them to an endocrinologist. However, there are no formal guidelines—we make referrals based on our own judgment.”*

(302)

### 3.4. Theme 2: Policy Implementers’ Self-Perceived Determinants of Policy Implementation

Participants’ perceptions of policies, and their implementation, indicated several determinants categorized either as self-perceived barriers or as self-perceived facilitators. These were further classified as individual, sociocultural, and structural self-perceived barriers or facilitators, based on the level at which they influenced the implementation process.

#### 3.4.1. Self-Perceived Barriers at Individual, Sociocultural, and Structural Levels

##### Self-Perceived Individual Barriers

Almost all participants reported having limited knowledge of childhood nutrition and physical activity needs/recommendations specifically in relation to the specific needs of children in need.

*“There is some knowledge. I think there should be more organized education.”*

(220)

Most participants indicated limited engagement in policies as a barrier to the successful implementation of nutrition policies. Limited engagement was enhanced by lack of incentives or opportunities to encourage active participation.

*“There’s a need to strengthen awareness and participation among all stakeholders……. For example, we recently organized a seminar on childhood obesity with a public health doctor at a school, involving pediatricians. Yet, attendance was minimal while information about these actions is broadly shared, participation is consistently insufficient.”*

(206)

About half of the participants noted low health/nutrition literacy and linguistic/language problems as obstacles to the implementation efforts.

*“Roma people lack health literacy regarding quality of life.”*

(104)

*“We are an intercultural school, and most children are not of Greek descent, struggling with the Greek language. They may want to participate, but they don’t understand some words, making it less effective.”*

(201)

Some participants also claimed that the school setting is often the only setting where children engage in physical activity, particularly children with disabilities or from low-socioeconomic-status backgrounds. As a result, the participants raised the issue of increased sedentary behaviors as an extra barrier to adopting healthy lifestyles.

*“These children (adolescents with special needs) do physical education at school, and it’s perhaps the only place where they get exercise. As far as I know, these children don’t participate in any extracurricular physical activities.”*

(204)

*“Unfortunately, many parents in our school (low SES school) don’t involve their children in extracurricular activities. After school, most kids stay at home playing on their phones, making physical education their only opportunity for movement and exercise.”*

(201)

Additionally, some participants noted family-related challenges, such as financial difficulties, heavy workloads, limited time for meal preparation at home, and poor utilization of maternal and child health services, specifically in Roma communities, as barriers to active participation in policy activities.

*“Parents, due to their extensive work schedule, are increasingly neglecting the quality of their children’s diet. Unfortunately, in recent years, I have seen far too many croissants, sodas, and chips as school meals for their children!”*

(201)

*“When they go to the hospital to give birth (Roma women)—since all of them deliver in public hospitals—the doctors and midwives inform them about the benefits and importance of breastfeeding. From our side (healthcare center), we also try to raise awareness and explain how beneficial it is. […] Few Roma women choose to breastfeed.”*

(221)

##### Self-Perceived Sociocultural Barriers

Most participants highlighted that sociocultural factors and child-specific characteristics posed significant challenges to policy implementation like food fixations in children with autism, culturally driven food preferences, and poor school attendance of Roma children.

*“Autistic children, for example, often have specific food fixations; they may only eat certain foods based on characteristics like color.”*

(107)

*“This brings us to the cultural differences in children’s food preferences. For instance, different ethnic groups prefer rice prepared in specific ways.”*

(108)

*“An issue the school faces in the Roma community is that children generally do not have consistent attendance or have poor school attendance. This makes it difficult to implement structured programs and maintain continuity in the curriculum.”*

(104)

Most participants highlighted social exclusion and geographical deprivation as key challenges that hinder effectiveness of the policies in addition to engagement in policy interventions as such.

*“The school is exclusively for Roma children, and located within their community makes it easier for them to attend. However, it isolates the children and doesn’t allow interaction with non-Roma children.”*

(104)

*“I would say that even though they (families in remote areas) do have access, the geographical dimension combined with the lack of education among the parents can often lead to a lack of access to healthcare for the children.”*

(102)

##### Self-Perceived Structural Barriers

Structural barriers were commonly highlighted by all participants. The majority of implementers, across all sectors, highlighted a lack of targeted policies for children in need or policies tailored to their needs.

*“There is no provision in the legislation for physical education classes to accommodate children in need, or children who are overweight, or children with special needs, or children who may not speak Greek well, or struggle to understand! It’s one-size-fits-all!”*

(101)

*“Here (child protection center), we collaborate with the Social Grocery Store precisely due to the lack of support needed to meet children’s needs with dignity!”*

(309)

Additionally, some study participants raised issues of unequal healthcare treatment and access for children in need as a challenge for effective and equal policy implementation.

*“For example, a 6-year-old child with autism might have the same basic health insurance rights as a typical child, but their access to the same service is not equal […] and may struggle significantly more in that setting if there are no sensory adaptations or strategies in place at the dental office… the experience could be overwhelming, ultimately depriving them of the care they need in a way that the typical child would not experience!”*

(102)

Most participants identified limited logistical equipment and facilities and insufficient human resources as barriers to policy implementation. For instance, as reported for Roma communities,

*“We must also consider the practicality: how can they prepare 100% healthy meals without electricity in the community (of Roma)? In the summer, there is no refrigeration to preserve food, and the feasibility of following dietary recommendations becomes questionable.”*

(215)

In addition, although children with disabilities attended “typical” schools, inadequate infrastructure left them feeling excluded.

*“If a child (with special needs or disabilities) is unable to engage in physical education or other lessons equally, due to the school lacking the necessary equipment or accommodation, simply placing them in a typical school is not enough!”*

(101)

As one of the participants noted, lack of personnel continues to hinder effective implementation of policies even when equipment is available.

*“No matter the tools, legislative requirements, or willingness, it is impossible to implement programs at the desired scale due to a lack of personnel and increased work overload (at a regional and municipal scale). There is an urgent need to hire professionals to adequately staff these services!”*

(206)

Moreover, the lack of effective monitoring mechanisms, such as in the school canteens or physical education lessons, was highlighted as an additional barrier to implementation by most study participants. About half of the participants referred to the lack of consistent or cross-sectoral policies, while some noted bureaucratic inefficiencies such as poor communication with authorities when needed.

*“The legislation (regarding food sold in school canteens) outlines the required products—we’re fully aware of that. However, I can say with absolute certainty that no one adheres to it! If we were to comply, we’d have to shut down our business, and there would be no canteens left!”*

(103)

*“Some actions do take place, but they are not continuous or widespread. They are often implemented in a fragmented manner by different organizations […] with no interaction or collaboration between them”*

(304)

*“For over six months, I have unsuccessfully tried to contact national authorities to discuss the inclusion of our association in national programs for obesity prevention!”*

(105)

Furthermore, some participants raised the issue of limited access to sports facilities for children in need, emphasizing that lack of supportive and enabling environments restricts their opportunities for physical activity. For instance, gyms often lack inclusive programs for children living with obesity or equal accessibility for those with disabilities, further reinforcing sedentary lifestyles and posing an additional barrier to effective and inclusive policies for these children.

*“There is a need to develop sports centers designed specifically for children with disabilities, as such facilities are limited…….and when available accessibility remains a challenge. To me, ensuring access to all sports facilities and offering adaptive sports programs tailored to these children is essential.”*

(305)

Self-perceived low governmental prioritization of promoting healthy lifestyles in school settings including overloaded curricula and reduced physical education hours was also raised by almost half of the participants.

*“Although theoretically, we receive guidelines from the relevant ministry for skill workshops in practice, there’s simply no time left for organized activities!”*

(201)

Finally, a senior staff member from an association for individuals living with obesity described lack of official recognition of obesity as a disease in Greece, describing it as a major barrier to effectively addressing the issue.

*“Obesity is not defined as a chronic disease and therefore people living with obesity cannot receive the benefits that should be provided to a chronic condition, e.g., prescription for dietitians, exercise, vouchers, prescription for certain healthy foods.”*

(105)

#### 3.4.2. Self-Perceived Facilitators at Individual, Sociocultural, and Structural Levels

##### Self-Perceived Individual Facilitators

Almost all participants perceived that willingness and awareness to support children in need was a major facilitator to the effective implementation of nutrition policies.

*“We don’t have anything organized here (health center). However, as pediatricians, we do attend informative seminars on obesity management from our university clinics in the private or public sector or hospitals (private and public). But this requires us to actively choose to attend these updates.”*

(302)

Some participants also perceived regular school attendance, particularly among Roma children, as a motivation and contributor to improved dietary habits. For example, Roma children attending school changed their nutritional habits during the school year.

*“You could easily distinguish between Roma children who attend school regularly and those who come sporadically. Children who initially brought sugar-sweetened beverages started bringing water instead!”*

(104)

Some participants stated that involving children in cooking activities served as experiential nutrition education that also motivated the children to healthier dietary habits.

*“We encourage and motivate the children (in the care center) to cook, always with caregivers and staff present, and they do it amazingly, especially some nationalities who see it as an enjoyable activity.”*

(108)

##### Self-Perceived Sociocultural Facilitators

Approximately half of the participants discussed participation in various community-led initiatives that contributed to promoting healthy eating habits and active lifestyles in children.

*“We have organized events and activities in various regions of Greece. Initially, we engaged with children, accompanied by a dietitian, to discuss nutrition in a conversational format, rather than through formal presentations or other conventional methods. We also incorporated numerous games, as visual and interactive play helps children retain the information more effectively.”*

(105)

##### Self-Perceived Structural Facilitators

Self-perceived structural facilitators were also frequently highlighted. Almost all of the participants perceived that integrating nutrition and physical education into school curricula supported healthy choices and active lifestyles for children, especially those in need. Few study participants also stressed that consistent long-term interventions led to better policy implementation by engaging children in need in a more effective way.

*“In this school (special education school), we also have nutrition/cooking workshops, and it is very important to us. We constantly address it in every lesson, discussing what we could replace with healthier alternatives. […] These children (adolescents) do physical education at school, and it’s perhaps the only place where they get exercise, given their limited participation in extracurricular physical activities.”*

(204)

*“If you implement a personalized school program and follow through until the end of the year, these children (with special needs) can experience significant changes […]. Some children who previously engaged in self-harming behaviors were started integrated into the class”!*

(101)

Some health sector participants stated that annual provision of governmental nutrition guidelines enhanced their knowledge and strengthened their ability to support children in need.

*“There are national guidelines and dietary recommendations that are sent regularly, at least once a year.”*

(301)

Approximately half of the participants described that provision of school meals served as a facilitator for effective implementation of nutrition policies. One participant also stated that provision of free school meals could potentially motivate Roma children to attend school regularly.

*“School meal provision was a motivation for Roma children to attend school […] We observed cases where, due to insufficient funding, the program would pause for a while, and during those times, attendance would drop and return upward again once the program started to run again.”*

(104)

Moreover, some participants specified that availability of appropriate logistical equipment and facilities enhanced productivity, and provided better support for children in need. One food sector participant emphasized the importance of involving specialists as a means of effective implementation. The participant stated that their role as a dietitian and administrator working in childcare units allows for provision of nutrition-tailored plans for children with health challenges or specific difficulties.

*“Last year, at a school where I worked, they brought in new equipment, except the usual soccer goalpost and basketball hoop that most schools have, a ping pong table. During breaks, all the students were eager to play. Adding more options like that or similar facilities would help a lot. Instead of just sitting around during breaks, students would have more opportunities for physical activity!”*

(203)

*“For example, if a child (in the care center) happened to have gastroenteritis, they would immediately inform me, and I would prepare the special menu for the same day. ….. clear instructions for the staff, and I had prepared guidelines for the facility’s caregivers for immediate response.”*

(107)

Furthermore, some participants underlined that free and equal access to sport facilities and healthcare centers for children in need, who often experience exclusion, served as a positive determinant for the health and physical activity.

*“When it is proven that a family is in need and the child, for mental health reasons, needs to engage in physical activity, the municipality can approve the child’s participation in a gym or other sports activities and support the membership fee.”*

(106)

## 4. Discussion

This study aimed to explore policy implementers’ knowledge and perceptions of the Greek national policy framework to prevent childhood obesity in children in need and self-perceived barriers and facilitators of its implementation process. To the authors’ knowledge, this is the first study in Greece to address this issue in depth by including a diverse sample of policy implementers from four key delivery systems across three diverse geographic regions of the country. These regions represent both urban and rural areas with parts of differing deprivation.

With regard to the policy framework, study participants, from the social protection, food, and education systems, reported knowledge of available policies and self-perceived implementation concerns. Additionally, participants expressed a self-perceived lack of targeted policy guidance for children in need, which is consistent with the literature [7,19]. A lack of available policies was noted by the health sector participants represented. This is of concern given the results of a systematic review indicating that many healthcare professionals were unaware of dietary guidelines, existing resources, or how to initiate weight management discussions, and it was concluded that understanding their perspectives is essential for designing effective interventions [19]. The findings of the current study indicated the school setting as the main environment in which policy implementers reported active participation in intervention policies including skill workshops, physical education curricula, food provision/assistance initiatives, and school canteens, confirming previous findings [11]. Schools are described in the literature as an ideal setting for health promotion initiatives and for reducing food insecurity in children [9,20,21,22,23], and, therefore, future national actions should capitalize on such findings.

The most prevalent self-perceived barriers of the study included limited knowledge and training among implementers in regard to child nutrition and physical activity guidelines, especially for children in need, and lack of incentives or opportunities for active participation, confirming previous results [19,24,25,26]. This is of concern specifically because it relates to teaching staff and their crucial role in the prevention of childhood obesity [7,26,27]. Similarly to other studies [8,24,28], inactivity outside the school setting was highlighted by the study participants, particularly affecting children with disabilities or those residing in disadvantaged areas and contributing to sedentary lifestyles. Previous research showed that vulnerable children, including those in the categories of children in need, face greater challenges in engaging with policy interventions [7,24,28,29,30,31,32], and have stressed the importance of tailored interventions to their needs as a means to promote health equity and inclusion [29,33]. For this reason, it is important that barriers associated with dimensions of social exclusion and geographical isolation (i.e., poor use of maternal and child health services and school attendance, resident status, low health/nutrition literacy, and language barriers) are taken into consideration when designing and implementing policies for the vulnerable population. This is particularly important for child populations included in the children in need categories because their lifestyle needs are likely to be exacerbated by a combination of types of vulnerability (e.g., disability, resident status, etc.) and of sociocultural characteristics.

Inappropriate infrastructure and facilities in schools, healthcare settings, and sport facilities not tailored to the needs of children in need, particularly children with disabilities, were commonly reported self-perceived barriers in this study, confirming previous results [24,26,28,31,32]. The study by Law et al. (2007) reported that children with disabilities faced particular challenges in inadequate environments, i.e., school, built environments, etc., raising issues of equity and wellbeing [28]. In the Greek context, this lack is often related to funding availability and possibly to a lack of structural prioritization to promoting healthy lifestyles in schools [11]. Administrative barriers were also raised by the study participants, including insufficient cooperation and coordination across sectors and stakeholders, combined with lack of personnel and overloaded curricula and limited physical education hours. This is something to be considered by the policymakers, as findings from a recent systematic review indicate that underfunded schools not only require financial support but also strong government-led policies and cross-sectoral coordination to overcome barriers to implementation [26]. It is worth pointing out that Roma communities in Greece, as in other European countries, face infrastructure deprivation, including lack of basic amenities such as electricity, running water, kitchen facilities, etc., [30,31,32] and should be considered in related community nutrition promotion activities. Studies have highlighted personal interest and commitment as key enablers of successful policy implementation [34,35], and that when implementers receive training in nutrition and physical education, they feel more confident to support children [26]. Policy implementers of this study raised the issue of a lack of training and guidance, but also showed strong willingness and motivation to support children in need by attending seminars or engaging in nutrition-related actions on their own initiative. This highlights the need for national action and provision of appropriate training (possibly certified as mean to encourage participation), training material/guidance addressing the needs of children in need, and standardized implementation tools to all professionals involved in the implementation process.

Another important finding of this study is that free school meals emerged as a self-perceived structural facilitator, with implementers emphasizing their importance for low-income families. Particularly for Roma children, free school meals can serve as an incentive for regular school attendance. Prior studies also showed that school food provision to all children not only promotes healthy eating habits but also enhances equity and reduces stigmatization among students [21,26]. In Greece, food provision and food assistance policies are available and have the potential to reach a large number of beneficiaries. However, concerns exist related to logistics and tendering procedures, fund availability, and poor uptake of the provision/assistance schemes, in addition to the lack of robust evidence of their effectiveness and impact on children in need [11].

This study has strengths and limitations. The qualitative approach used is the most suitable for capturing knowledge and perceptions of policy implementers in depth. Additionally, a combination of the semi-structured questionnaire guide and a sufficient number of interviewers from four key delivery systems, across three different regions in Greece, strengthens the results of the study and possibly allows generalizability of the results to children in need in other regions of the country. However, due to the context-specific nature of the study, the results may not be generalized to different populations. Also, certain topics with potential influence on the implementation process, i.e., the role of industry, were not explored as part of the study and/or were not raised by the study participants, despite the flexibility of the semi-structured approach; hence, their influence cannot be determined. In addition, qualitative methods are susceptible to information bias due to the data collection dependency on the interviewer’s skills to extract the information efficiently. To minimize this error, an experienced health psychologist and behavioral scientist with extensive expertise in qualitative research conducted a training session with team members to guarantee consistency and high-quality results, as well as to ensure inter-rater reliability during the data collection.

## 5. Conclusions

Personal initiative emerged as one of the main self-perceived determinants for active participation in the implementation process; however, self-perceived structural issues like available infrastructure and lack of cross-sectional collaboration hinder such efforts despite recent governmental efforts in the country. The findings of this study have direct and immediate implications for the development and implementation of policies tackling the obesogenic environment in children in need, both in Greece and other European countries with similar contexts. Potential actions could include development of certified mandatory trainings of evidence-based training material/guidance and provision of standardized implementation tools to facilitate quality standards with the potential to strengthen the motivation, capacity, and ability of the policy implementers to actively and efficiently participate in the implementation process.

## Figures and Tables

**Table 1:** Descriptive characteristics of the policy implementers participating in the study.

Number of Participants, N = 25	
Age (years)	
Median (IQR)	42 (34, 53)
Duration of current employment (years)	
Median (IQR)	9 (4, 15)
Gender N (%)	
Female	18 (72%)
Male	7 (28%)
Delivery System N (%)	
Education	7 (28%)
Food	7 (28%)
Health	5 (20%)
Social Protection	6 (24%)
Educational Level N (%)	
Doctoral studies	1 (4%)
Postgraduate studies	17 (68%)
Undergraduate studies	4 (16%)
Post-secondary education	1 (4%)
High School	2 (8%)
Region N (%)	
Attica	8 (32%)
Thessaly	8 (32%)
Crete	9 (36%)

## Data Availability

The data presented in this study are not available due to privacy.

## Supplementary Materials

The following supporting information can be downloaded at: https://www.mdpi.com/article/10.3390/nu17162629/s1, Table S1a–d: Topic guides for conducting semi-structured interviews with policy implementers (for respective delivery systems), Table S2: Job position and respective work system of the policy implementers participating in the study (N = 25).
