# Supplementary material for: Addressing Childhood Obesity in Children in Need in Greece: Policy Implementers’ Knowledge, Perceptions and Lessons for Effective Implementation
Source: Nutrients. 2025 Aug 14;17(16):2629. doi: 10.3390/nu17162629 (PMC12388959; doi:10.3390/nu17162629)
Supplement: Supplementary file 1 [file nutrients-17-02629-s001.zip › nutrients-3768428-supplementary.pdf]

Supplementary Materials

Table S1a-S1d. Respective topic guides for conducting semi-structured interviews with policy implementers.

Table S1a. Topic guides for conducting semi-structured interviews with policy implementers in the education system.

**Topic guides for conducting semi-structured interviews with policy implementers in the education system.**
1. What do you think is the importance of the school environment in preventing childhood obesity?
2. In what ways does the school promote and encourage: (a) healthy eating and (b) physical activity, both, in and out of school/ school schedule?
3. Do you think that the existing curriculum and skills workshops promote nutritional literacy, physical activity, and limiting screen time?
4. Do you believe that the entire educational community (teachers, professors, physical education, professors, principals) has the required knowledge, support, and tools to be a role model for children in promoting healthy eating, physical activity, and limiting screen time?
5. Are there actions and initiatives carried out by the State or other institutions (e.g. E.U.) to promote healthy eating and physical activity at school?
6. Do you have clear directives from the Ministry of Education (e.g., existence of guidelines) as well as the necessary technical equipment for the implementation of the Physical Education class?
7. Do you think that the Physical Education class actually helps children to be physically active?
8. Do you think that the Physical Education class gives equal opportunities and motivates all children to be physically active (e.g., boys - girls, children with overweight and/or obesity)?
9. Do you think that a cross-sectoral nutritional policy (involving health, education, food, and social protection sectors) at the national level would be effective in preventing childhood obesity (as opposed to fragmented actions that currently take place in some regions/schools)?
10. What are your suggestions for actions and initiatives within the school, that you would expect to be taken concerning what we have discussed today, so that the school would be more effective in promoting: (a) healthy eating and (b) physical activity for all children, and especially for children in need?
11. What are your suggestions for changes to the current legislative framework that you would expect to be made, concerning what we have discussed today, so that the school would be more effective in promoting: (a) healthy eating and (b) physical activity for all children, and especially for children in need?

Table S1b. Topic guides for conducting semi-structured interviews with policy implementers in the food system.

**Topic guides for conducting semi-structured interviews with policy implementers in the food system.**
**Questions for the canteen managers/staff**
1. What kind of food is usually available in the canteen?
2. Is there a specific legislative framework that determines the availability of food in school canteens? Are you aware of the content of this legislative framework?
3. What would you suggest so that, on the one hand, we promote healthier choices within the school, and on the other hand, you can make a profit as a business?
**Questions for people in charge of feeding children in child protection institutions/ facilities**
4. Is there a specific legislative framework that defines the feeding/ nutrition of children in social protection institutions/ facilities?
5. Who is responsible for designing the weekly menu residing in the institution?
6. What kind of food is usually available in the institution?
**General questions**
7. "Food insecurity" refers to the limited or uncertain availability or access to sufficient, safe, and nutritious food. Do you think that children in need are at greater risk of being in a situation of "food insecurity"? Do you think that the current legislative framework for food labeling contributes to the adoption of healthy dietary choices for children?
8. What changes to the current legislative framework could ensure access to healthier food for all children (e.g., tax reduction on specific foods such as fruit, vegetables, and dairy for school canteens/child protection institutions, implementation of policies to protect children from the trade in foods high in saturated fatty acids, trans fatty acids, free sugars, and/or salt?
9. Do you think that a cross-sectoral (including health, education, food, and social protection sectors) national nutrition policy would be effective in preventing overweight and childhood obesity for all children? For what reason? What do you think it should include?
10. With what actions could the State, the municipality, and/or the school contribute to the promotion of healthy eating for all children?

Table S1c. Topic guides for conducting semi-structured interviews with policy implementers in the health system.

**Topic guides for conducting semi-structured interviews with policy implementers in the health system**
1. Are there organized actions and initiatives by the State, through the health system, for the prevention of childhood obesity (e.g., awareness-raising actions in Health Centers on healthy eating and active living)?
2. Is there provision in the current legislative framework for periodic assessment of children's development and for providing counseling for families, to prevent overweight and childhood obesity (e.g., promotion of healthy diet and physical activity)?
3. Is there provision in the current legislative framework for providing counseling and support for children and their families identified with overweight and/or obesity?
4. Do you think that all health professionals (e.g., pediatricians, health visitors, nurses) have sufficient support, training, tools, and information on childhood obesity issues?
5. Do you think that all health professionals are adequately informed about childhood nutrition guidelines?
6. Is there provision in the current legislative framework for counseling on healthy eating and physical activity during pregnancy?
7. Do you believe that all children have equal access (and equally easy access) to the health care system, including children in need (e.g., the existence of local primary healthcare centers to provide care to children with disabilities or local primary healthcare centers near Roma communities)?
8. What actions and initiatives by the State could contribute to the prevention of childhood obesity through the health system?
9. Do you think that a cross-sectoral nutritional policy (involving health, education, food, and social protection sectors) at a national level would be effective in preventing childhood obesity (as opposed to fragmented actions that currently take place in some regions/schools)?
10. What actions and/or changes to the current legislative framework would you expect to see concerning what we have discussed today?

Table S1d. Topic guides for conducting semi-structured interviews with policy implementers in the social protection system.

**Topic guides for conducting semi-structured interviews with policy implementers in the social protection system.**
1. Are there any actions and initiatives carried out by the State or other organizations to promote healthy eating and physical activity for children in need (e.g., actions to promote healthy eating in child protection institutions, actions to promote breastfeeding in Roma communities)?
2. Do you think that the State's actions and/or provisions, through the Social Protection sector (e.g., financial support, Social Grocery scheme) contribute to the prevention of overweight and childhood obesity for children in need?
3. Is there a specific legislative framework that defines the nutrition of children in child protection structures and/or children in institutions? If so, who is responsible for implementing the legislative framework?
4. Do you think that social protection professionals have sufficient support, training, tools, and awareness on issues related to childhood overweight and obesity?
5. What changes in the current legislative framework could support children in need to adopt behaviors such as healthy eating and active living to prevent overweight and childhood obesity?
6. Do you think that a cross-sectoral (including health, education, food, and social protection sectors) national nutrition policy would be effective in preventing overweight and childhood obesity for all children? For what reason? What do you think it should include?
7. What actions and/or changes in the current legislative framework would you expect to be taken, concerning what we have discussed today?

Table S2. Employment position and respective delivery system of the policy implementers participating in the study (N=25).

ID	Delivery System	Employment position
	**Education**	
101		Special Education Teacher, public primary school[1]
104		Psychologist, primary and secondary schools in Roma community
201		Special Education Teacher, public primary school[1]
202		Feeding Speech Therapist, daycare and creative activity center for people with disabilities[1]
203		Educator & Nurse, Special Vocational Education and Training Laboratories for adolescents
303		Physical Activity Teacher, public primary school
304		Teacher, public primary school in Roma community
	**Health**	
102		Occupational Therapist, special therapies center
206		Senior regional public health specialist
221		Nurse, Health Center for Roma
301		Health Professional, Health Center[1] (& former volunteer for migrant children)
302		Pediatrician, Mother-Child and Adolescent Protection Station, Health Center[1]
	**Food**	
103		Canteen Manager, public primary school[1]
107		Dietitian & Nutrition Administrator of Unaccompanied Minors Asylum Seekers
108		Administrator, Reception Center for Unaccompanied Refugee Minors
204		Cook & Educator in Special Vocational Education and Training Laboratories for adolescents
226		Canteen Manager, middle and high school[1]
308		Cook, Children Protection Center
320		Cook & Educator, Child Protection Center
	**Social Protection**	
105		Senior employee at a national obesity association
106		Social Worker, Mental Health Center
215		Psychologist in Health Center for Roma (supporting Roma families)

220	Social worker, Child and Family Support Center
305	Social Worker, Special Education School
309	Social Worker, Child Protection Center

[1]Children in need included
